# Vomiting as a Symptom and Transmission Risk in Norovirus Illness: Evidence from Human Challenge Studies

**DOI:** 10.1371/journal.pone.0143759

**Published:** 2016-04-26

**Authors:** Amy E. Kirby, Ashleigh Streby, Christine L. Moe

**Affiliations:** Hubert Department of Global Health, Rollins School of Public Health, Emory University, Atlanta, GA, United States of America; Virginia Polytechnic Institute and State University, UNITED STATES

## Abstract

**Background:**

In the US, noroviruses are estimated to cause 21 million cases annually with economic losses reaching $2 billion. Outbreak investigations frequently implicate vomiting as a major transmission risk. However, little is known about the characteristics of vomiting as a symptom or the amount of virus present in emesis.

**Methodology and Principal Findings:**

Emesis samples and symptomology data were obtained from previous norovirus human challenge studies with GI.1 Norwalk virus, GII.2 Snow Mountain virus, and a pilot study with GII.1 Hawaii virus. Viral titers in emesis were determined using strain-specific quantitative RT-PCR. In all four studies, vomiting was common with 40–100% of infected subjects vomiting at least once. However, only 45% of subjects with vomiting also had diarrhea. Most of the emesis samples had detectable virus and the mean viral titers were 8.0 x 10^5^ and 3.9 x 10^4^ genomic equivalent copies (GEC)/ml for GI and GII viruses, respectively (p = 0.02). Sample pH was correlated with GII.2 Snow Mountain virus detection.

**Conclusions and Significance:**

Half of all subjects with symptomatic infection experienced vomiting and the average subject shed 1.7 x 10^8^ GEC in emesis. Unlike shedding through stool, vomiting is more likely to result in significant environmental contamination, leading to transmission through fomites and airborne droplets. This quantitative data will be critical for risk assessment studies to further understand norovirus transmission and develop effective control measures. The correlation between sample pH and virus detection is consistent with a single site of virus replication in the small intestine and stomach contents becoming contaminated by intestinal reflux. Additionally, the frequency of vomiting without concurrent diarrhea suggests that epidemiology studies that enroll subjects based on the presence of diarrhea may be significantly underestimating the true burden of norovirus disease.

## Introduction

Noroviruses are estimated to cause 21 million cases of acute gastroenteritis every year in the US [1]. Although most cases of AGE are self-limiting, it is estimated that up to 71,000 cases are hospitalized and 510–800 deaths occur annually [1]. Two genogroups of norovirus, GI and GII, are responsible for the majority of human disease [2]. These genogroups are further divided into 9 and 21 genotypes, respectively. A meta-analysis of published norovirus outbreaks observed that GI viruses are more frequently associated with environmental transmission and GII viruses are more likely to be associated with person-to-person transmission and healthcare-associated outbreaks [3]. Currently, there is no specific treatment for norovirus, though a bi-valent GI.1, GII.4 vaccine is in development [3].

Noroviruses are transmitted through the fecal-oral route via consumption of contaminated food or water, contact with contaminated surfaces (fomites), and person-to-person [4–10]. Outbreak investigations have implicated vomiting events as a significant contributor to transmission risk, either by contamination of surfaces or creation of aerosols that can be inhaled through the mouth [8, 11–19]. Marks et al. investigated a large outbreak in an elementary school and found that risk of norovirus illness increased with each vomiting event that occurred in a classroom [14]. Proximity to a vomiting event has also been shown to correlate with attack rates [13, 19]. In several instances, the contamination from the initial vomiting event continued to cause infections for several days and, in some cases, after cleaning [8, 15, 19].

Despite the clear role of vomiting in transmission, there is very little data on viral loads in emesis. An investigation of norovirus outbreaks among rafters in the Grand Canyon detected GI norovirus in a vomitus sample [20]. In samples collected during a GI.1 Norwalk virus human challenge trial, 56% of emesis samples had detectable virus and the median titer was 4.1x10^4^ gEq/ml [21]. There is no data available for the more common GII noroviruses.

The goal of this study is to provide quantitative data describing the frequency of vomiting and virus titers during norovirus infection. We analyzed vomiting data from two GI.1 Norwalk virus challenge studies, a GII.2 Snow Mountain virus challenge and a pilot study with GII.1 Hawaii virus. Virus titers were determined by RT-qPCR in the archived emesis samples from these studies. The availability of this data will facilitate accurate transmission risk estimation and provide an evidence base for the development of appropriate control measures, such as disinfection after a public vomiting event.

## Methods

### Human Challenge Studies

The emesis samples for this study are archived specimens from three previously published human challenge studies (GI.1 Norwalk 8fIIb and GII.2 Snow Mountain Virus) [22–24] and one pilot study (GII.1 Hawaii Virus). Dosing schemes for each study are given in Table 1. Briefly, healthy adult subjects were admitted to the hospital research unit on Day 0. After providing a pre-challenge stool sample, the subjects were challenged with safety-tested norovirus inoculum that was suspended in water ([23, 24] and Hawaii virus (pilot)) or injected into raw oysters [22]. The subjects were kept in isolated hospital rooms for 5 days post-challenge. Symptoms were assessed by study staff at least twice each day. Anti-emetics were offered to subjects in all studies if excessive vomiting posed a risk of dehydration. During the inpatient period, all stools and emesis samples were collected for virus testing, and the time of collection was recorded. No subjects reported vomiting after discharge from the research unit., nor did any uninfected subjects experience vomiting.

**Table 1:** Human Challenge Studies Contributing Samples to This Study.

Study	Strain	Dose (GEC ^a^)	Delivery	# Enrolled	# Infected ^b^	# Ill ^c^ (%)	# With Vomiting (%)	Reference
Genogroup I								
1	GI.1 Norwalk 8fIIb	≤ 1 x 10^4^	Oyster	54	15	10 (67)	6 (40)	[22]
2	GI.1 Norwalk 8fIIb	≤ 6.5x10^7^	Water	13	10	10 (100)	8 (80)	[24]
Genogroup II								
3	GII.2 Snow Mountain	≤ 1.2x10^5^	Water	15	9	9 (100)	6 (67)	[23]
4	GII.1 Hawaii	8.0x10^6^	Water	2	2	2 (100)	2 (100)	This study

^a^ Genomic equivalent copies.

^b^ Defined as detection of norovirus RNA in at least one stool or ≥ 4-fold rise in anti-norovirus IgG in serum.

^c^ Defined as diarrhea (alone) or one or more vomiting episodes plus one of the following: abdominal cramps, nausea, fever (oral temperature ≥37.6°C), myalgia, chills, fatigue, or headache.

Emesis and stool samples were weighed, aliquoted into sterile containers and frozen at -80°C. For emesis samples, weight was used as a proxy for total volume with 1 g equal to 1 ml. Samples collected within 15 min of each other were combined and treated as a single specimen occurring at the earlier recorded time.

Infection was defined as detection of norovirus RNA in at least one stool specimen or ≥ 4-fold increase in anti-norovirus IgG in serum. Diarrhea was defined as ≥ 3 unformed stools or ≥400 g unformed stool in a 24 hr period. Viral gastroenteritis was defined as detection of norovirus RNA in stool, and diarrhea (alone) or one or more vomiting episodes plus one of the following: abdominal cramps, nausea, fever (oral temperature ≥37.6°C), myalgia, chills, fatigue, or headache.

All challenge studies were approved by the Emory University Institutional Review Board (studies 1 and 2) [22, 24] or the University of North Carolina Institutional Review Board (studies 3 and 4) [23]. All subjects provided written consent for study participation and future use of study specimens. Studies 1 and 2 are registered at clinicaltrials.gov (trials NCT00674336 and NCT00313404, respectively). Studies 3 and 4 were completed prior to mandatory trials registration.

### RNA Isolation

Emesis (50% vol/vol) and stool (20% vol/vol) suspensions were prepared in sterile, molecular-grade water. For emesis specimens, only the liquid phase of the specimen was used to maintain testing consistency. Virus particles were separated from organic debris by phase extraction with an equal volume of Vertrel XF (DuPont, Wilmington, DE). After incubating for 2 hours at 4°C, the sample was centrifuged for 10 minutes at 9400 x g. RNA was isolated from 140 μl of the aqueous phase using the QiaAmp Viral RNA mini kit (Qiagen, Valencia, CA) following the manufacturer’s instructions. Isolated RNA was stored at -20°C until testing.

### Quantification of Viral Copies

Viral titers were determined using quantitative reverse transcription polymerase chain reaction (qRT-PCR) using previously described strain-specific primers and probes and the OneStep RT-PCR kit (Qiagen, Valencia, CA) [25, 26]. The Snow Mountain virus primers were used to test Hawaii virus samples, as the primer and probe target sequences are identical between the two strains. In vitro-transcribed Norwalk or Snow Mountain RNA standards were used to generate standard curves, which were used to estimate genomic equivalent copies (GEC) per well. The assays were performed on a BioRad CFX96 Real-Time PCR Detection System (Bio-Rad Laboratories, Hercules, CA) with the cycling program: 50°C for 32 min, 95°C for 10 min, 45 cycles of 95°C for 15 sec followed by 56°C for 1 min. The final titers were reported as GEC/ml for emesis and GEC/g for stool.

### pH Determination

The pH of completely thawed emesis samples was determined using pHydrion plastic pH strips, 0.0 to 6.0 (Micro Essential Laboratory, Brooklyn, NY) and colorPHast pH strips, 5–10 (EMD Millipore, Billerica, MA).

### Statistical Analyses

Comparisons between studies and between genogroups were assessed using the Student’s t-test. ANOVA was carried out in GraphPad Prism 6 (GraphPad Software, Inc., La Jolla, CA).

## Results

The samples used in this study were archived from previous norovirus human challenge trials (Table 1). Of the 25 subjects infected with Norwalk virus, 20 became ill and 14 vomited at least once. The earliest vomiting episode was 20 hrs post-challenge. In both of the Norwalk virus studies, the challenge dose was treated prior to administration; the oysters were subjected to high-pressure hydrostatic processing and the spiked groundwater was stored at room temperature for varying times. Thus, it is not possible to know the exact dose of active virus in each challenge, only the upper limit which is reported in Table 1. Fifteen subjects were challenged with one of three doses of Snow Mountain virus, 9 became ill and 6 vomited. There was no relationship between dose and vomiting (data not shown). The pilot infectivity study of Hawaii virus only had two subjects, but both became ill and vomited. Due to the small sample size, only descriptive results are presented for Hawaii virus and the data was not included when comparing results between genogroups. There were a total of 57 archived emesis samples available from 22 subjects for this study. None of the uninfected subjects in any of the studies experienced vomiting.

Vomiting is very common in symptomatic norovirus infection. Among subjects infected with Norwalk virus, 70% of symptomatic subjects experienced at least one vomiting event (Table 1). Similarly, 72% of symptomatic subjects infected with one of the GII viruses experienced vomiting. Vomiting was of short duration; the mean time between first and last vomiting events ranged from 2.0 to 10.8 hr (Table 2). The number of vomiting events ranged from 1 to 7, with 32% of subjects only vomiting once. On average, subjects produced 658.7 ml and 845.0 ml of emesis over the course of their illness with GI.1 Norwalk virus or GII.2 Snow Mountain virus, respectively. Among subjects infected with GI norovirus, 57% of vomiting subjects also met the study definition of diarrhea (≥3 loose stools or ≥400g loose stool in 24 hr). Among subjects infected with GII norovirus, 50% of vomiting subjects also had diarrhea. There were no statistically significant differences in vomiting frequency, duration, volume or diarrhea frequency between GI Norwalk virus (studies 1 and 2) and GII Snow Mountain virus (study 3) infections.

**Table 2:** Characteristics of Vomiting as a Symptom.

Study	N	Vomiting Events/ Subject		Mean Duration^a^ (hrs)(SEM^e^)	Mean Volume/Subject (ml)(SEM^e^)	Frequency of Concurrent Diarrhea^b^
		Min, Max	Mode			
1	6	1, 7	1	4.9 (3.1)	489.2 (206.6)	67%
2	8	1, 5	1,3	9.7 (6.2)	785.8 (111.5)	50%
All GI	14	1, 7	1	7.7 (3.7)	658.7 (111.9)	57%
3	6	1, 4	1,2	2.0 (0.8)	845.0 (226.7)^c^	50%
4	2	4, 6	-	10.8 (2.1)	1439.0 (ND)^d^	50%

^a^ For subjects with only one vomiting event, a duration of 1 minute was assigned.

^b^ Diarrhea was defined as ≥ 3 loose stools or ≥ 400g loose stool produced in 24 hours.

^c^ Two subjects were missing volume data and are not included in this analysis.

^d^ One subject was missing volume data and is not included in this analysis.

^e^ Standard error of the mean.

**Fig 1:**
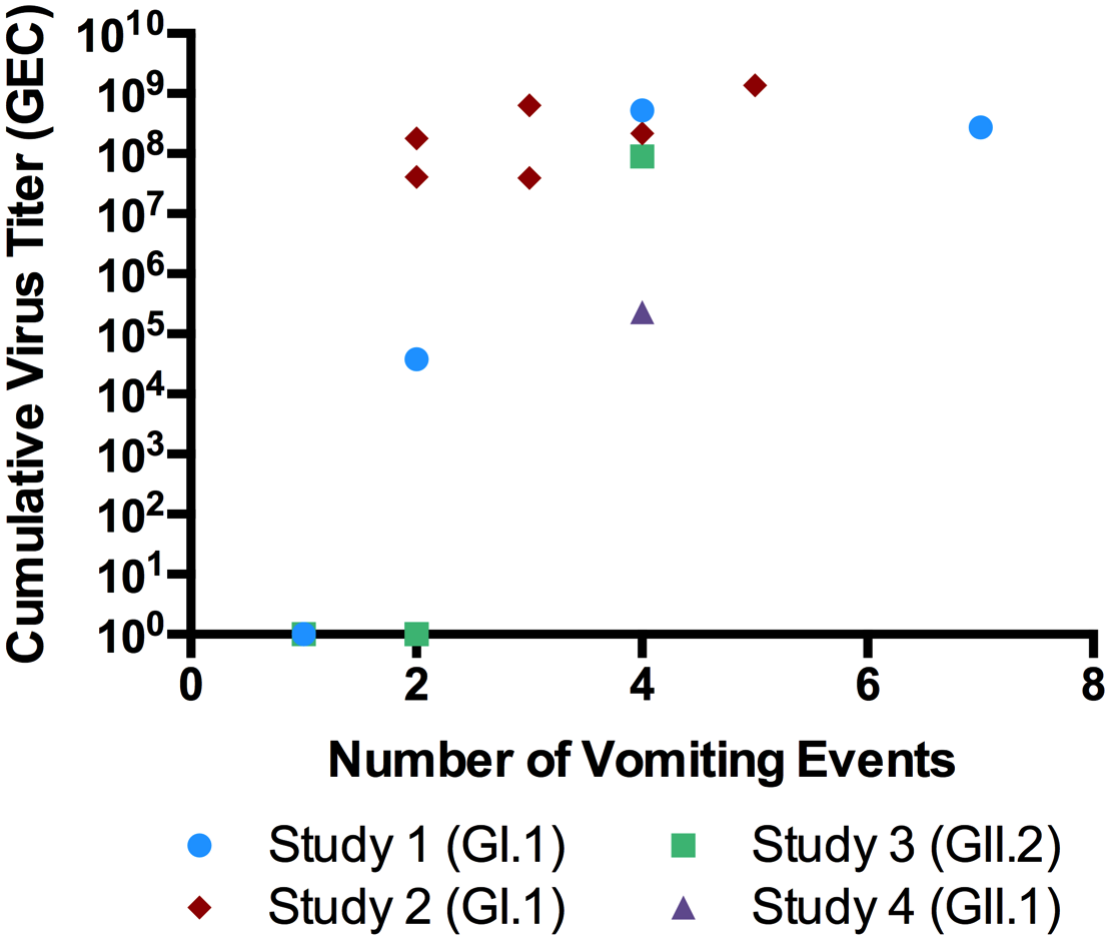
Subjects With More Vomiting Events Have Higher Cumulative Virus Titers. Cumulative virus titers are calculated by multiplying each sample’s virus titer by the sample volume and summing across all of the emesis samples from a subject. Vomiting events occurring within 15 minutes were considered a single event and the samples were combined for analysis. Each point represents a unique challenge subject, except at the baseline where multiple points overlap. Seven subjects vomited once and virus was not detected in any of those samples. Two subjects vomited twice and both samples were negative for virus.

Most subjects who experienced vomiting had at least one sample with detectable norovirus (Table 3). Of the subjects who only vomited once, none had detectable virus in their emesis sample. In contrast, when subjects experienced multiple vomiting events, the first vomiting event was often norovirus-positive (57%, Fig 1). There was no difference in viral titers between GI.1 Norwalk virus and GII.2 Snow Mountain virus samples (8.0x10^5^ GEC/ml vs. 1.6x10^5^ GEC/ml, p = 0.36). Samples from subjects infected with Hawaii virus had lower mean virus titers but a higher frequency of positive samples. To assess total viral shedding in emesis, cumulative shedding was calculated by multiplying the sample virus titer by the sample volume in ml and summing the resulting value across all positive samples for a subject. Overall, the cumulative virus shedding per subject was high (1.8x10^8^ GEC +/- 7.8x10^7^, Norwalk and Snow Mountain viruses only). The cumulative shedding titers for subjects infected with Norwalk or Snow Mountain viruses were similar, but the cumulative shedding during Hawaii virus infection was 2–3 logs lower than the other viruses (2.3x10^5^). However, it should be noted that only one subject infected with Hawaii virus had sample volume data available.

**Table 3:** Norovirus Titers in Emesis.

Study	# Subjects with Emesis Specimens	# Emesis Specimens	% Subjects with ≥ 1 Positive Emesis	% Positive Samples	Sample Mean Titer^c^ (GEC^d^/ml)(SEM^e^)	Subject Mean Cumulative Shed (GEC^d^)(SEM^e^)
1	6	16	50%	63%	5.8x10^5^ (2.6x10^5^)	1.3x10^8^ (9.1x10^7^)
2	8	20	75%	90%	9.2x10^5^ (3.1x10^5^)	3.1x10^8^ (1.7x10^8^)
All GI	14	36	64%	78%	8.0x10^5^ (2.2x10^5^)	2.3x10^8^ (1.0x10^8^)
3	4^a^	8	25%	38%	1.6x10^5^ (4.5x10^4^)	1.8x10^7^ (1.8x10^7^)
4	2	13	100%	92%	5.0x10^3^ (2.7x10^3^)	2.3x10^5^ (ND)^b^

^a^ Two subjects with vomiting excluded due to missing samples.

^b^ One subject excluded due to missing volume data.

^c^ Of samples with detectable virus.

^d^ Genomic equivalent copies.

^e^ Standard error of the mean.

**Fig 2:**
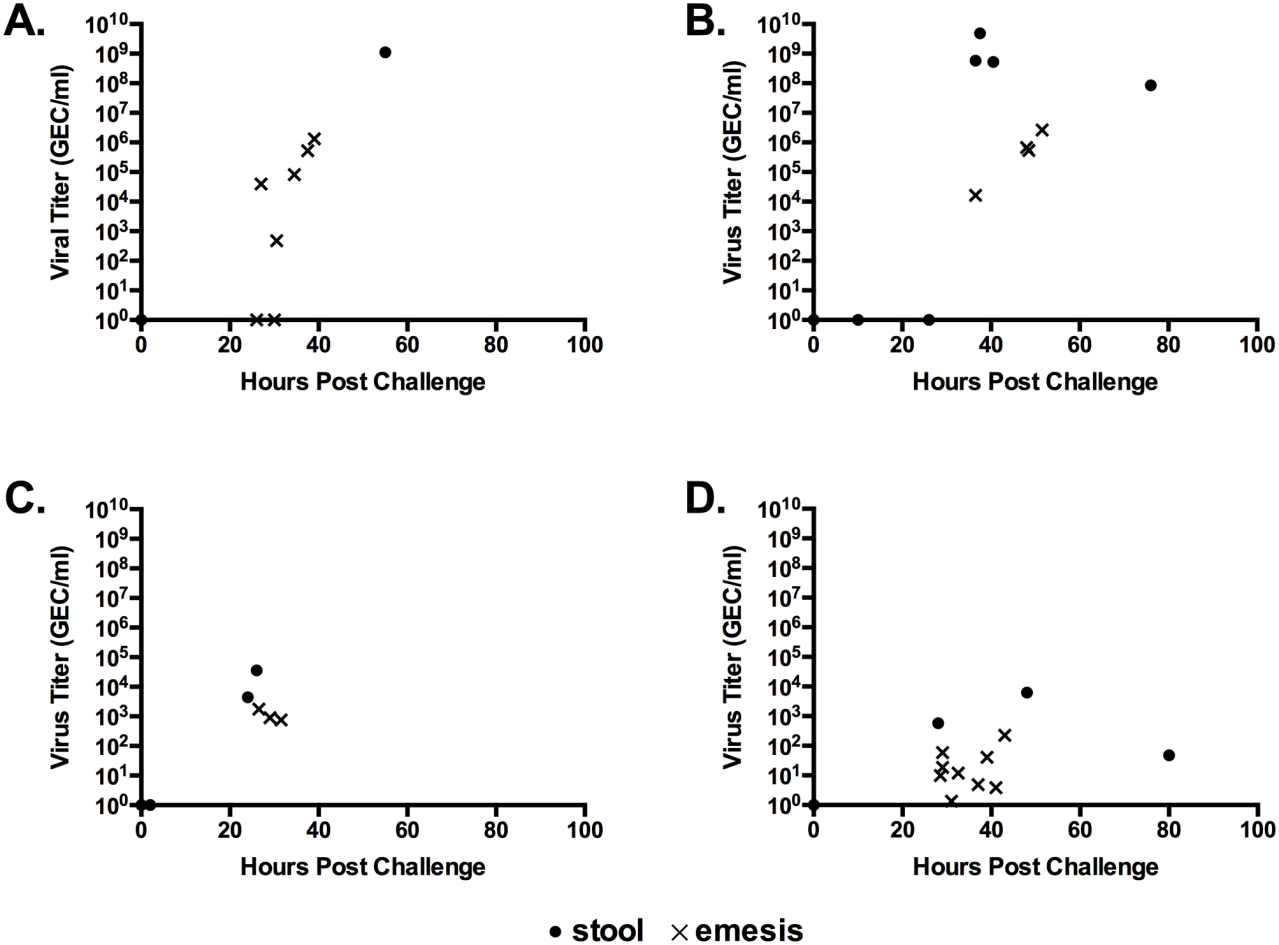
Viral Titers in Emesis and Stool from Representative Challenge Subjects. Selected subjects provided stool and emesis samples during the first 100 hours after challenge, allowing comparisons of stool and emesis viral titers in the same timeframe. Panels A and B are from subjects in study 1 infected with GI.1 Norwalk virus. Panel C is a subject in study 3 infected with GII.2 Snow Mountain virus. Panel D is a subject in study 4 infected with GII.1 Hawaii virus.

The relationship between virus titers in emesis and stool from representative subjects is shown in Fig 2. In all four studies, viral titers in emesis were lower than those in stool. Most subjects did not have stool and emesis samples during the same time period, making statistical analysis of correlations in titer problematic. However, viral titers in emesis tended to increase at the same rate as the titers in stool, though not to the same magnitude.

The pH of stomach contents can vary based on diet, time of day and prolonged vomiting [27]. To assess the impact of this change on viral titers, the pH of the emesis samples was determined. There was a significant difference in the pH of samples where virus was detected and samples without any virus detected (one-way ANOVA, p<0.0001). There was a trend towards higher sample pH in samples with detectable virus. There was poor correlation between sample pH and viral titer.

## Discussion

It is clear from outbreak and challenge studies that vomiting is the signature symptom of norovirus infection, which is colloquially known as the “winter vomiting disease.” However, vomiting has been largely overlooked in the quantitative studies of norovirus disease. This is the first report of vomiting frequency and viral titers from human challenge studies with GI and GII noroviruses. In these challenge studies, vomiting was more prevalent than diarrhea as a symptom and norovirus-infected subjects shed large amounts of virus through vomiting. There was no difference in viral titers in emesis samples between subjects infected with GI.1 Norwalk and subjects infected with GII.2 Snow Mountain virus. (Table 3), in contrast to the titers in stool where subjects with Norwalk virus infections had higher titers [25]. Even with sample volume taken into account, there was no difference in mean cumulative virus shedding between GI and GII infections. Given the low infectious dose for Norwalk virus [21, 28], a single vomiting event could contain sufficient virus to infect over 150,000 individuals. However, the true infectivity may be over-estimated due to the detection of non-infectious virus in the RT-qPCR analysis. At this time, there is no in-vitro infectivity assay for human norovirus.

Simulated vomiting events have shown that a single vomiting event can contaminate a large area (7.8 m^2^) and produce potentially infectious aerosols [29, 30]. A recent study found that norovirus can survive in simulated emesis in suspension and on surfaces for up to 42 days [31]. Thus, it is critical to respond swiftly and appropriately to a vomiting event with thorough cleaning and disinfection with a chlorine-based disinfectant. Additionally, isolation and anti-emetic treatment should be implemented at the first episode of vomiting to limit the potential for environmental contamination and transmission. This is particularly important because viral titers tend to increase with each additional vomiting episode (Fig 1).

This is the first report of results from a human challenge study with GII.1 Hawaii virus. The viral titers in emesis and stool (Table 2 and Fig 2) were several logs lower than those reported for other challenge inocula [21, 25], yet the subjects experienced severe vomiting and diarrhea. These results suggest that Hawaii virus may be more virulent than the other inoculum strains. However, this was a pilot study with only two subjects. More research is needed to further elucidate the clinical course and pathogenesis of Hawaii virus.

**Fig 3:**
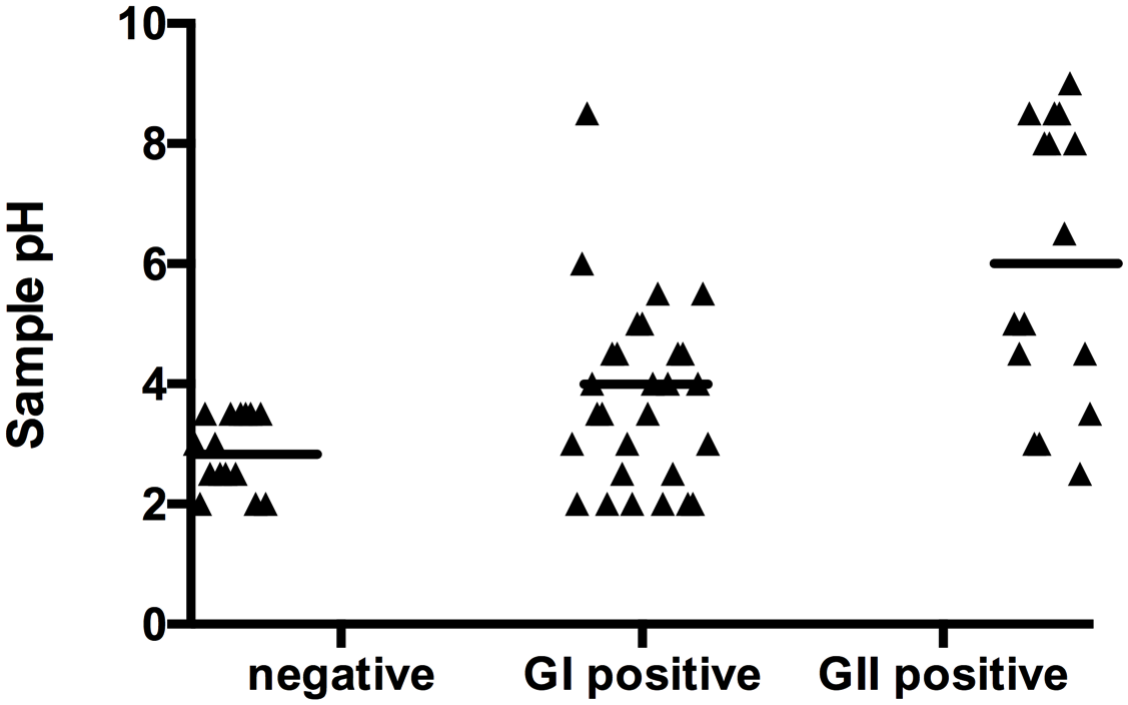
Correlation Between Sample pH and Detection of Virus. GI positive samples are from studies 1 and 2, both GI.1 Norwalk virus. GII positive samples are from studies 3 and 4, GII.2 Snow Mountain virus and GII.1 Hawaii virus, respectively. Negative samples is a compilation of negative samples from all four studies.

The site of norovirus replication within the host is not known, but the virus has been shown to bind to duodenal tissue [32]. The results of this study are consistent with a single site of virus replication in the duodenum or liver, as suggested by Karst and Wobus [33]. For subjects with overlapping stool and emesis specimens, the virus titers in emesis were consistently lower than those in stool, but the rate of titer increase in the two sample types was similar (Fig 2), as would be expected from a shared replication site. Additionally, there was a trend towards higher pH in samples with detectable virus (Fig 3), consistent with reflux of duodenenal contents into the stomach. Like prolonged vomiting, diet and time of day can also cause an increase in stomach pH [27] and may have increased the pH of early vomiting events, making the detection of significant changes in pH more unlikely. However, emesis samples were produced during the day and night and there was no correlation between time of production and sample pH (data not shown). Diet was not controlled or recorded during the challenge studies, so it is not possible to assess its impact on sample pH.

Many outbreak and epidemiology studies of norovirus define a case based on the presence of diarrhea. However, in this study, nearly half of subjects who experienced vomiting did not have concurrent diarrhea (Table 2). Our findings suggest that the diarrhea-only case definition will result in a significant underestimate of the true prevalence of norovirus disease and misclassification bias in case-control studies. Future studies should include vomiting as part of the case definition to reflect the full clinical presentation of symptomatic norovirus illness.
